# Association of multiple serum minerals and vitamins with metabolic dysfunction-associated fatty liver disease in US adults: National Health and Nutrition Examination Survey 2017–2018

**DOI:** 10.3389/fnut.2024.1335831

**Published:** 2024-03-18

**Authors:** Peisen Guo, Jiahui Yu

**Affiliations:** The Center of Gastrointestinal and Minimally Invasive Surgery, Department of General Surgery, The Third People’s Hospital of Chengdu, The Affiliated Hospital of Southwest Jiaotong University, Chengdu, China

**Keywords:** vitamins, minerals, joint effect, MAFLD, NHANES

## Abstract

**Background:**

Despite the rapid increase in the global prevalence of Metabolic Dysfunction-Associated Fatty Liver Disease (MAFLD), there are no approved therapeutic drugs for MAFLD yet. Nutrient supplementation might mitigate the risk of MAFLD. It is more typical for individuals to consume multiple nutrients simultaneously. However, the studies exploring the combined effects of multiple nutrients on MAFLD are limited. This study aimed to investigate the relationship between both individual nutrients and their combined influence on the risk of MAFLD.

**Methods:**

Data were obtained from National Health and Nutrition Examination Survey (NHANES), and 18 types of nutrients were considered in this study. Logistic regression analysis was performed to evaluate the correlation between single nutrients and the risk of MAFLD. The Least Absolute Shrinkage and Selection Operator (LASSO) regression analysis was performed to pinpoint the most relevant nutrient associated with the risk of MAFLD. Subsequently, both Weighted Quantile Sum (WQS) regression and Quantile g-computation (Qgcomp) were used to assess the combined effects of multiple nutrients on the risk of MAFLD.

**Results:**

A total of 3,069 participants were included in this study. LASSO regression analysis showed that Se, α-tocopherol, and γ-tocopherol exhibited a positive association with the risk of MAFLD. In contrast, the serum levels of Co, P, α-cryptoxanthin, LZ, and trans-β-carotene were inversely associated with the prevalence of MAFLD. When Se and two types of vitamin E were excluded, the WQS index showed a significant inverse relationship between the remaining 15 nutrients and the risk of MAFLD; α-cryptoxanthin showed the most substantial contribution. Similarly, Qgcomp suggested that the combined effects of these 15 nutrients were associated with a lower risk of MAFLD, with α-cryptoxanthin possessing the most significant negative weights.

**Conclusion:**

This study suggested that the complex nutrients with either a low proportion of Se, α-tocopherol, and γ-tocopherol or without them should be recommended for patients with MAFLD to reduce its risk.

## 1 Introduction

In 2020, an international panel of experts proposed the concept of Metabolic Dysfunction-Associated Fatty Liver Disease (MAFLD) (1). In contrast to Non-Alcoholic Fatty Liver Disease (NAFLD), MAFLD is defined using different diagnostic criteria (1) and is often depicted as a hepatic insulin-resistant disease, which is predominantly instigated by the dysfunction of lipid metabolism (2). The global prevalence of MAFLD is estimated to be 50.7% in overweight or obese individuals (3) and 33.87% in overweight or obese children and adolescents (4). Studies suggest that MAFLD is significantly correlated with adverse health outcomes, such as cardiovascular disease, hepatic events, extrahepatic malignancies, and renal disease (5). A distinguishing characteristic of MAFLD is excessive hepatic fat accumulation, which can be accurately and non-invasively assessed using FiberScan with Vibration-Controlled Instantaneous Elastography (VCTE) (6).

Hepatic steatosis is typically induced by unhealthy diets rich in fructose, saturated fats, and cholesterol. As there are no approved pharmacological treatments for MAFLD, the primary management strategies include physical activity and dietary modifications (2). Previous studies have indicated that a healthy lifestyle can decrease the risk of metabolic syndrome (7). Specific dietary nutrients can mitigate the symptoms of MAFLD and decrease its risk. For instance, vitamins C and D3 can modulate the gut microbiota and bile acid metabolism, thereby alleviating MAFLD symptoms (8). Moreover, a study indicated a positive correlation between the serum vitamin D concentration and a reduced risk of MAFLD (9). Similar findings have been reported regarding the dietary intake of vitamin K (10). On the other hand, a mendelian randomization study showed an elevated risk of MAFLD associated with an increase in liver iron concentration (11). Multiple factors, such as oxidative stress-induced lipotoxicity and cellular senescence, contribute to the pathogenesis and progression of MAFLD (12). Additionally, inflammatory responses accelerate the development of MAFLD (13). A study reported that several components of traditional Chinese herbal medicine could inhibit liver inflammation and alleviate liver damage (13). Therefore, supplementing the antioxidant or anti-inflammatory nutrients might have similar effects.

Numerous studies have focused on the correlations between individual nutrients and MAFLD. However, due to the intricate interactions among various nutrients, evaluating the effect of a single nutrient might lead to skewed results. For instance, the higher intake of calcium (Ca) and phosphorous (P) might affect the absorption of Mg, inducing various metabolic diseases (14). Moreover, a higher molar ratio of zinc (Zn) to copper (Cu) can cause Cu deficiency, leading to the dysregulation of metabolic factors (15). Notably, patients with liver diseases have dysregulated absorption and metabolism of several nutrients (16). Therefore, when the patients with MAFLD are supplemented with nutrients, the ratio among various nutrients is different from that of normal people. Mixed effects are commonly reported in studies assessing the effects of environmental pollution on human health (17–19). Correspondingly, individuals consume a combination of nutrients; however, the studies examining the combined effects of multiple nutrients are limited. Therefore, this study aimed to evaluate the association between individual serum nutrient levels and the combined effects of multiple serum vitamins and minerals on the risk of MAFLD. Meanwhile, the ratio among various nutrients was also determined. The evaluation was performed using Weighted Quantile Sum (WQS) regression and Quantile g-computation (Qgcomp) regression models on the data obtained from the National Health and Nutrition Examination Survey (NHANES) 2017–2018.

## 2 Materials and methods

### 2.1 Study population

The data of participants included in this study were obtained from the NHANES 2017–2018 survey. Among the 9,254 participants in the NHANES survey, 5,494 individuals went through the liver elastography examination. The participants below the age of 18 were subsequently excluded (*n* = 748). Among the remaining subjects, the detection of serum cobalt (Co) concentration was performed for 3,133 individuals aged 40 years and above. Moreover, 53 participants who could not be definitively diagnosed with MAFLD, and 11 participants who responded with “refuse” or “do not know” during questionnaire interviews about alcohol use and education level were excluded. Ultimately, this study included a total of 3,069 participants.

### 2.2 Definition of MAFLD

As per the international panel of experts, MAFLD diagnosis is based on hepatic steatosis coupled with one of the following three conditions: overweight/obesity, type 2 diabetes mellitus, or metabolic dysregulation (20). Metabolic dysregulation is defined as the conditions with at least two of the following indicators (20): (a) waist circumference ≥ 102/88 cm in Caucasian men and women; (b) blood pressure ≥130/85 mmHg or specific drug treatment; (c) plasma triglycerides ≥1.70 mmoL/L or specific drug treatment; (d) plasma high density lipoprotein-cholesterol (HDL-C) <1.0 mmol/L for men and <1.3 mmol/L for women or specific drug treatment; (e) prediabetes, such as fasting glucose level 5.6 to 6.9 mmol/L, or HbA1c 5.7 to 6.4%; (f) homeostasis model assessment (HOMA)-insulin resistance (IR) score ≥2.5; and (g) plasma high-sensitivity C-reactive protein (hs-CRP) level >2 mg/L. According to the existing literature, the condition with a controlled attenuated parameter (CAP) score of ≥248 dB/m is diagnosed as liver steatosis (21); the CAP score is determined using the FibroScan^®^ model 502 V2 Touch.

### 2.3 Covariates

The covariates considered in this study included age, sex, race, education level, family income, physical activity (PA), and smoking and drinking status. The patients were grouped into ≤60 and >60 based on age and Mexican American, Other Hispanic, Non-Hispanic White, Non-Hispanic Black, and other races based on race. Based on education level, the patients were divided into less than high school, high school, and more than high school groups (22). Family income was assessed using the poverty income ratio (PIR), and the patients were classified into low-income (PIR < 1.30), middle-income (1.3 ≤ PIR < 3.5), and high-income (PIR ≥3.5) groups (23). Based on smoking status, the patients were divided into non-smoker (smoked less than 100 cigarettes in their lifetime) and smoker (smoked more than 100 cigarettes in their lifetime) groups. Based on drinking status, they were divided into non-drinker (those who had never consumed alcohol or had not consumed alcohol in the past 12 months) and drinker (those who had consumed alcohol in the past 12 months) groups. According to the literature (24), PA was calculated using Eq. 1.

(1) $$\begin{array}{c} PA\;\left(\mathrm{MET}-h/wk\right)=\mathrm{metabolic}\;\mathrm{equivalent}\;\left(\mathrm{MET}\right)\times \mathrm{weekly}\;\mathrm{frequency}\\ \;\times \mathrm{duration}\;\mathrm{of}\;\mathrm{each}\;\mathrm{physical}\;\mathrm{activity} \end{array}$$

The three variables in Eq. 1 were obtained from the PA questionnaire on the NHANES website. Then, based on PA, the patients were classified into low PA (<1MET-h/week), moderate PA (1–48 MET-h/week), and high PA (>48 MET-h/week) groups.

### 2.4 Statistical analyses

All the statistical analyses were performed using the R software (4.2.2). A total of 18 nutrients, including vitamin A, vitamin C, vitamin D, α-tocopherol, γ-tocopherol, lutein + zeaxanthin (LZ), α-carotene, trans-β-carotene, α-cryptoxanthin, β-cryptoxanthin, lycopene, iron (Fe), selenium (Se), Ca, Co, sodium (Na), potassium (K), and P were included in the analysis in this study. If the serum concentration of a specific substance was below the limits of detection (LOD), the value was represented as LOD divided by √2. For the 3,069 participants, any missing data, including house income, PA, smoking status and 18 nutrients, was imputed using grouping-based (age, gender, and race) median of the available samples for continuous variable, as well as the grouping-based mode for category variable, including drinking status. Variables, such as house income, PA, and smoking status, were then classified into category variables. Furthermore, the serum contents of the 18 nutrients were natural log-transformed. Continuous variables were represented as the means ± SD (standard deviation), and a student’s *t*-test was used to compare differences between the two groups. Categorical variables were expressed as frequencies (percentages), and the differences between groups were compared using a chi-square test.

Initially, two logistic regression models, including model 1 and model 2, were used to explore the association between single nutrients and MAFLD. Model 1 was an unadjusted model, while, model 2 was adjusted for age, gender, race, education level, family income, smoking status, drinking status, and PA. The least absolute shrinkage and selection operator (LASSO) regression analysis was used to identify the nutrient most relevant to the risk of MAFLD (25). The data of 18 nutrients were standardized and centralized and then subjected to LASSO regression analysis. The dose-response relationship between MAFLD and various vitamins and minerals was evaluated using a restricted cubic spline (RCS) model. The combined effects of multiple minerals and vitamins were evaluated using WQS and Qgcomp regression models. Notably, the RCS model, WQS regression model and Qgcomp regression model were adjusted for age, gender, race, education level, family income, smoking status, drinking status and PA. For the WQS regression, 40% of the participants were randomly selected as training dataset, and 60% of the participants served as validation datasets. The bootstrap value was set to 1,000 in the parameter estimations. The Qgcomp regression estimated the joint effects of increasing every nutrient simultaneously by one quantile (26). The Qgcomp regression was conducted without bootstrap to estimate the weight of each nutrient and with bootstrap set to 500 to estimate the marginal odds ratio of the joint effect. Finally, a sensitivity analysis was performed to validate the robustness of the results, and the data were re-analyzed after removing all the participants with missing values.

## 3 Results

### 3.1 Baseline characteristics of study participants

As per the definition of MAFLD, 1,968 individuals were diagnosed with MAFLD among the 3,069 participants (Table 1). The average age of all the participants was approximately 60 years. The incidence of MAFLD was higher in males than females (52.0% vs. 45.0%). There were no significant statistical discrepancies between the MAFLD and non-MAFLD groups in terms of education level, family income, smoking status, PA and drinking status. Moreover, the patients with MAFLD were more likely to have higher aspartate aminotransferase (AST), gamma-glutamyl transferase (GGT) and alkaline phosphatase (ALP). Strikingly, as compared to non-MAFLD patients, the serum concentrations of γ-tocopherol (1.43 ± 0.52 vs. 1.23 ± 0.54), α-tocopherol (3.38 ± 0.31 vs. 3.35 ± 0.31) and Se (0.89 ± 0.13 vs. 0.87 ± 0.14) were elevated in the MAFLD patients; all the differences were statistically significant (*p* < 0.05).

**Table 1:** Basic characteristics of participants.

Characteristic	MAFLD		*p*
	No, *N* = 1,101	Yes, *N* = 1968	
Age, year	60.38 ± 12.21	60.39 ± 11.17	0.987
Age, %			0.665
≤60	555 (50.4%)	976 (49.6%)	
>60	546 (49.6%)	992 (50.4%)	
Sex, %			<0.001
Female	605 (55.0%)	944 (48.0%)	
Male	496 (45.0%)	1,024 (52.0%)	
Race, %			<0.001
Mexican American	97 (8.8%)	290 (14.7%)	
Other Hispanic	81 (7.4%)	206 (10.5%)	
Non-Hispanic White	386 (35.1%)	692 (35.2%)	
Non-Hispanic Black	310 (28.2%)	420 (21.3%)	
Other Race	227 (20.6%)	360 (18.3%)	
Education level, %			0.386
<High school	222 (20.2%)	432 (22.0%)	
High school	254 (23.1%)	466 (23.7%)	
>High school	625 (56.8%)	1,070 (54.4%)	
Family income, %			0.315
Low	255 (23.2%)	433 (22.0%)	
Middle	514 (46.7%)	975 (49.5%)	
High	332 (30.2%)	560 (28.5%)	
Smoking status, %			0.786
Never smoking	607 (55.1%)	1,075 (54.6%)	
Smoking	494 (44.9%)	893 (45.4%)	
Physical activity, %			0.294
High	305 (27.7%)	530 (26.9%)	
Middle	493 (44.8%)	844 (42.9%)	
Low	303 (27.5%)	594 (30.2%)	
Drinking status, %			0.904
Drinker	707 (64.2%)	1,268 (64.4%)	
Non-drinker	394 (35.8%)	700 (35.6%)	
Ln ALT, U/L	2.83 ± 0.47	3.02 ± 0.50	<0.001
Ln AST, U/L	3.01 ± 0.35	3.02 ± 0.35	0.178
Ln GGT, IU/L	3.06 ± 0.66	3.31 ± 0.67	<0.001
Ln ALP, IU/L	4.32 ± 0.29	4.37 ± 0.28	<0.001
Ln LZ, μmol/L	−1.06 ± 0.59	−1.18 ± 0.57	<0.001
Ln lycopene, μmol/L	−0.50 ± 0.60	−0.51 ± 0.54	0.799
Ln β-cryptoxanthin, μmol/L	−1.98 ± 0.86	−2.08 ± 0.83	0.002
Ln trans-β-carotene, μmol/L	−1.11 ± 0.97	−1.42 ± 0.88	<0.001
Ln α-cryptoxanthin, μmol/L	−3.08 ± 0.60	−3.21 ± 0.56	<0.001
Ln α-carotene, μmol/L	−2.69 ± 1.06	−2.94 ± 0.99	<0.001
Ln γ-tocopherol, μmol/L	1.23 ± 0.54	1.43 ± 0.52	<0.001
Ln vitamin A, μmol/L	0.60 ± 0.30	0.62 ± 0.29	0.103
Ln α-tocopherol, μmol/L	3.35 ± 0.31	3.38 ± 0.31	0.014
Ln vitamin D, nmol/L	4.24 ± 0.45	4.19 ± 0.46	0.001
Ln vitamin C, μmol/L	3.82 ± 0.73	3.71 ± 0.71	<0.001
Ln Se, μmol/L	0.87 ± 0.14	0.89 ± 0.13	<0.001
Ln Fe, μmol/L	2.68 ± 0.42	2.66 ± 0.39	0.180
Ln Co, nmol/L	1.13 ± 0.48	1.03 ± 0.47	<0.001
Ln P, mmol/L	0.13 ± 0.14	0.11 ± 0.14	<0.001
Ln K, mmol/L	1.41 ± 0.09	1.41 ± 0.09	0.556
Ln Na, mmol/L	4.94 ± 0.02	4.95 ± 0.02	0.582
Ln Ca, mmol/L	0.84 ± 0.04	0.84 ± 0.04	0.759

Data are represented as proportions (%) for categorical variables and as mean ± SD for continuous variables. Student’s *t*-test and chi-square test were used for continuous and categorical variables to explore the difference between groups. LZ, lutein + zeaxanthin; ALT, alanine aminotransferase; AST, aspartate aminotransferase; GGT, gamma glutamyl transferase; ALP, alkaline phosphatase.

### 3.2 Impacts of individual nutrients on MAFLD

**Figure 1:**
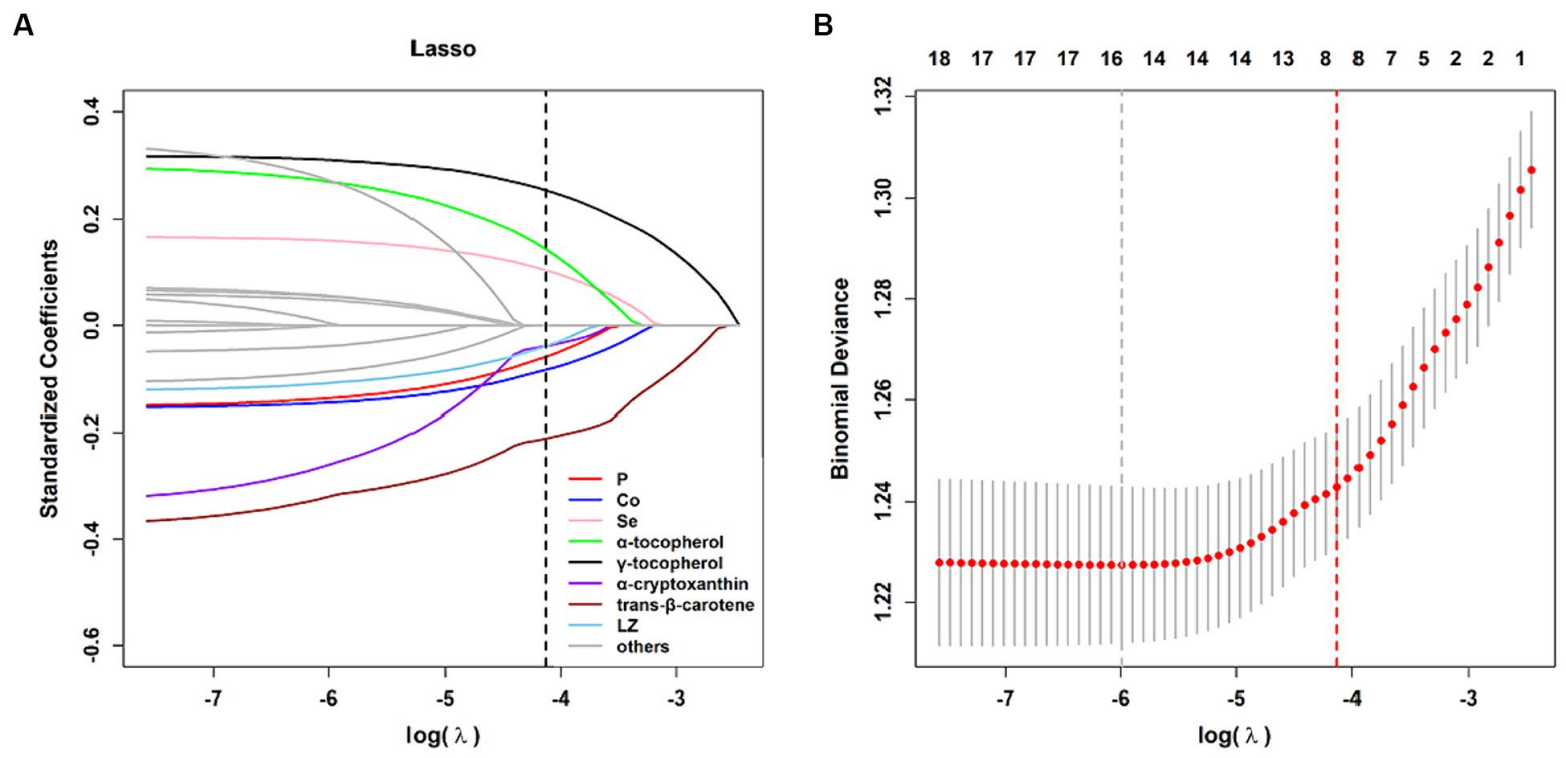
Results of LASSO regression model. **(A)** Plot for the coefficients of LASSO regression. **(B)** Ten-fold cross-validation for LASSO regression to select the optimal penalty coefficient.

Logistic regression analysis was performed to assess the effects of individual nutrients on the risk of developing MAFLD (Supplementary Table S1). For minerals, the serum levels of Co (OR = 0.503, 95% CI: 0.389–0.648) and Fe (OR = 0.704, 95% CI: 0.564–0.877) in only the Q4 group exhibited an inverse correlation with the risk of MAFLD in model 2. In both the Q3 and Q4 groups, the serum Se concentration was associated with the prevalence of MAFLD. Vitamins and carotenoids, including vitamin C, vitamin D, LZ, β-cryptoxanthin, trans-β-carotene, α-cryptoxanthin and α-carotene, were associated with a reduced risk of MAFLD; the serum trans-β-carotene levels in the Q4 group showed the highest efficacy (OR = 0.350, 95% CI: 0.277–0.442). Conversely, the serum levels of γ-tocopherol and α-tocopherol were positively correlated with the risk of MAFLD. LASSO regression analysis revealed the most critical variables for the risk of MAFLD. A 10-fold cross-validation was performed to select the optimal penalty parameter (*λ* = 0.016) (Figure 1A). As the parameters λ achieved this value, the Se, α-tocopherol, γ-tocopherol, P, Co, α-cryptoxanthin, trans-β-carotene, and LZ remained in the model (Figure 1B), indicating that the former three nutrients were positively correlated with the risk of MAFLD, while the latter five nutrients showed the opposite trend.

### 3.3 Dose-response relationship between nutrients and MAFLD

The RCS results revealed that an increase in the serum levels of Co, Fe, lycopene, LZ, trans-β-carotene, vitamin C, vitamin D, α-carotene, α-cryptoxanthin, and β-cryptoxanthin was significantly associated with the lower risk of MAFLD (Supplementary Figures S1A–J). Conversely, an increase in the serum levels of Se and γ-tocopherol was correlated with a higher risk of MAFLD, particularly the γ-tocopherol level (Supplementary Figures S1K,L). Furthermore, an increase in the serum levels of Ca, Na, Vitamin A, and α-tocopherol could moderately increase the risk of MAFLD (Supplementary Figures S1M–P), while an increase in the serum levels of K and P could marginally mitigate the risk of MAFLD (Supplementary Figures S1Q,R).

### 3.4 Combined effects of multiple nutrients on MAFLD

The WQS regression showed statistically significant results in both the positive and negative constrains, suggesting that the mixture of nutrients might not affect MAFLD; this result was confirmed by Qgcomp regression analysis (Supplementary Table S2).

**Figure 2:**
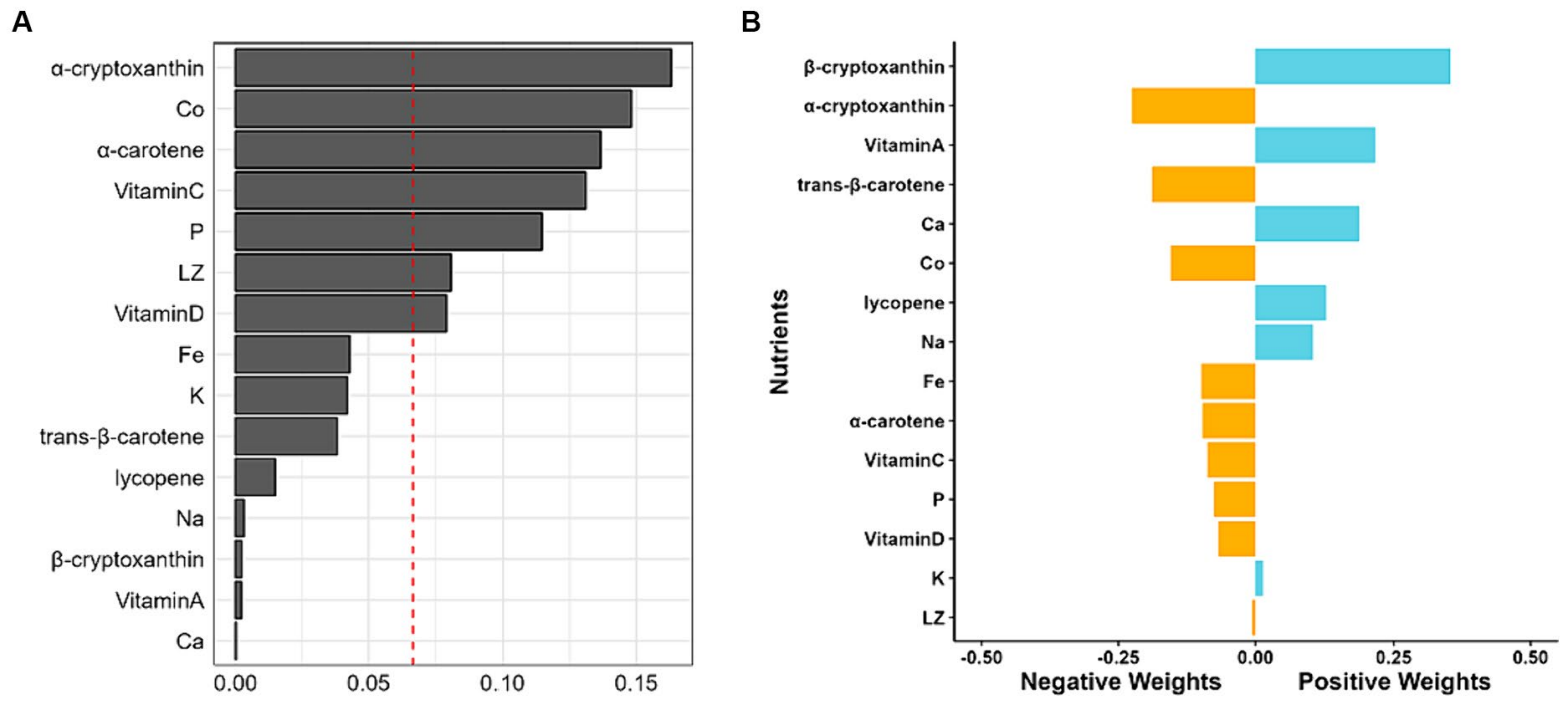
Weights of each nutrient in combined effect. **(A)** Weights calculated with WQS regression with negative constrain. **(B)** Weights of each nutrient revealed by Qgcomp, with the negative weights being statistically significant.

Given the significant contribution of five nutrients, including α-tocopherol, γ-tocopherol, Se, Na and Ca, to the positive relationship between the WQS index and MAFLD (Supplementary Figure S2A), WQS regression analysis was performed after consecutively excluding any one, two, or three of the five nutrients. As shown in Supplementary Figures S2B–D, the combined exclusion of Se, α-tocopherol and γ-tocopherol diminished the positive correlation between the WQS index and MAFLD. A 10% increase in the serum levels of the remaining 15 nutrients correlated with a 26.3% reduced risk of MAFLD (OR = 0.736; 95% CI: 0.682–0.794) (Table 2), among which, α-cryptoxanthin, Co, α-carotene, vitamin C, P, LZ, and vitamin D significantly contributed to this combined effect (Figure 2A). Concurrently, the Qgcomp regression analysis also revealed that the combined effect of these 15 nutrients was negatively associated with the risk of MAFLD (Marginal OR = 0.792; 95% CI: 0.732–0.856) (Table 2). The weights of these nutrients in the combined effect calculated using Qgcomp are shown in Figure 2B.

**Table 2:** Combined effect of 15 nutrients.

Methods	OR (95% CI)
WQS positive	1.046 (0.986, 1.109)
WQS negative	0.736 (0.682, 0.794)
Qgcomp	0.792 (0.732, 0.856)

Combined effects were evaluated using WQS regression with positive and negative constrains and Qgcomp. WQS, weighted quantile sum; Qgcomp, quantile-g-computation; OR, odds ratio; 95% CI, 95% confidence interval. Both WQS regression and Qgcomp were adjusted for age, gender, race, education level, smoking status, drinking status, physical activity and poverty income ratio.

### 3.5 Sensitivity analysis

The data of participants containing at least one missing value was discarded leaving a total of 1,974 subjects eligible for further analysis. Initially, both the WQS and Qgcomp regression analyses were employed to assess the combined effects of the 18 nutrients. The WQS results revealed both positive and negative constraints to be statistically significant (Supplementary Table S3), and the Qgcomp results suggested that the combined effect of the 18 nutrients did not confer any benefits towards the risk of MAFLD (Supplementary Table S3). Interestingly, excluding Se and two types of vitamin E resulted in retaining statistical significance in only the negative WQS index (Supplementary Table S4). Concurrently, the combined effects evaluated using the Qgcomp regression model displayed an inverse correlation with the risk of MAFLD (Supplementary Table S4).

## 4 Discussion

At present, MAFLD poses a significant threat to global health. Therefore, a cross-sectional study was conducted to investigate the combined effects of multiple nutrients on the risk of MAFLD. Three different statistical models, including LASSO regression, WQS regression, and Qgcomp models, consistently highlighted that the serum levels of Se, α-tocopherol, and γ-tocopherol were positively correlated with the risk of MAFLD. Conversely, all three methods demonstrated that the serum levels of Co, P, α-cryptoxanthin, LZ, and trans-β-carotene were inversely related to the risk of MAFLD. Subsequently, the result showed that the combined effects of 15 nutrients in serum, excluding Se, α-tocopherol, and γ-tocopherol, were advantageous in reducing the incidence of MAFLD. These findings suggested that a complex nutrient supplement, which can lower the serum levels of Se, α-tocopherol and γ-tocopherol, should be advised for patients with MAFLD to mitigate its risk.

Typically, people are simultaneously exposed to countless minerals and vitamins in day-to-day life, resulting in potential interactions among various nutrients. The over-supplementation of one nutrient can disrupt the absorption of others. Hence, examining the health impacts of a single nutrient on human wellness might yield skewed results. Previous studies have illustrated that co-exposure to a mixture of nutrients, including β-carotene, vitamin A, vitamin D, vitamin C, α-tocopherol, folate, vitamin B6, and vitamin B12, could diminish the risk of all-cause mortality in diabetic patients (27). Furthermore, the combined effects of vitamin C, vitamin B9, and vitamin B12 could limit the risk of metabolic disorders (28). Researchers demonstrated that adherence to a mineral-based nutrient pattern, which refers to the inclusion of multiple vitamins and minerals in an individual’s daily diet, was associated with healthier metabolic factors (29). They also suggested that increasing the plant-based nutrients, such as vitamins D, B6, B3, C, B1, E, etc., were associated with a lower risk of metabolic syndrome (30). These results demonstrated the beneficial effects of combined exposure to multiple nutrients, which were consistent with the findings in the current study. Moreover, a cocktail of 11 antioxidant nutrients, including Se and α-tocopherol, could decrease the risk of specific cardiovascular diseases (CVDs), with Se making the most substantial contribution (31). However, the findings in the current study indicated that a complex nutrient solution, including Se and two types of vitamin E, might not present beneficial effects on mitigating the risk of MAFLD. Interestingly, a low serum α-tocopherol level was correlated with a lower likelihood of overweight/obesity. Consequently, it was speculated that this phenomenon might be related to the age of participants, all of whom were over 40 years of age. Additionally, some unhealthy lifestyle choices or other unseen factors might diminish or even reverse the beneficial effects of certain nutrients. For instance, β-carotene supplementation in smokers might amplify the incidence of cardiovascular disease and mortality, as well as the risk of lung cancer (32, 33).

Vitamin E exerts its health benefits primarily through its antioxidant and anti-inflammatory properties (34, 35). Both the α-tocopherol and γ-tocopherol can modulate mitochondrial oxidative metabolism to ameliorate Alzheimer’s disease (36). Furthermore, γ-tocopherol could inhibit the inflammatory response and oxidative stress to enhance wound healing in diabetic rats (37), and α-tocopherol could also suppress inflammatory cytokines, such as tumor necrosis factor-α (TNF-α), interleukin (IL)-18, IL-12, and IL-6 (38). Although the onset and progression of metabolic disorders and fatty liver diseases are partially attributed to and concurrent with oxidative stress and inflammatory factors (39–41), Sabina reported that an increase in circulating α-and γ-tocopherol levels were positively associated with the risk of metabolic syndrome (42). This finding aligns with the current study results. This phenomenon might be partially explained by metabolic syndrome inhibiting the metabolism of α-tocopherol (43). Furthermore, the health effects of vitamin E and its metabolites are largely dependent upon the individual’s lifestyles, such as smoking status and alcohol consumption (44). Gut dysbiosis induced by MAFLD might also affect the serum levels of vitamin E and its metabolites (45). In conclusion, further research is needed to elucidate the specific mechanisms of this.

Although Se has been recognized as an indispensable trace element, its effects remain a subject of debate. Several studies have suggested that Se possesses numerous health-enhancing effects, such as reducing fasting insulin (46), fasting plasma glucose (47) and serum CRP levels (48). In obese individuals, Se can decrease body fat mass and augment lean body and muscle mass (49). However, researchers have discerned a positive correlation between plasma Se levels and fasting plasma glucose levels in men (50). Furthermore, high dietary Se intake is positively correlated with HOMA-IR in obese/overweight adults (51). Shao et al. (52) reported that an elevated Se status could increase the risk of diabetes in individuals aged 40 years. Therefore, combined with the current study results, it was suggested that middle-aged or elderly individuals might have elevated Se levels, increasing the risk of metabolic-dysfunction-associated diseases.

Both WQS and Qgcomp models revealed that α-cryptoxanthin played the most significant role in a negative correlation between the complex nutrients and the risk of MAFLD. To the best of our knowledge, though α-cryptoxanthin could induce mammalian phase 2 proteins to shield cells from damage by oxidants and electrophiles (53), few studies have reported beneficial effects of α-cryptoxanthin. Notably, numerous studies have focused on another cryptoxanthin known as β-cryptoxanthin, which is an antioxidant and a retinoid precursor that can mitigate the risk of NAFLD and other lifestyle-related diseases (54); however, it has deleterious effects on smokers and drinkers (55). In the current study, after excluding Se and two types of vitamin E, the Qgcomp demonstrated that β-cryptoxanthin had the most substantial positive weights. Moreover, WQS results indicated that β-cryptoxanthin slightly contributed to the negative correlation between the WQS index and MAFLD. These findings conflicted with the results of single nutrient analysis in RCS results. Similarly, Qgcomp results indicated that excluding Se and two types of vitamin E changed serum K from the largest positive weight to a slightly negative weight. These phenomena might be attributed to potential interactions among various nutrients. Further studies should investigate the contrasting effects of α-and β-cryptoxanthin on MAFLD.

The current study also suggested that trans-β-carotene played a pivotal role in decreasing the risk of MAFLD by ameliorating the indicators related to metabolic disorders. It may function as an antioxidant to decrease serum CRP levels and individual inflammatory burden (56). Researchers showed that a mixture of carotenoids was inversely associated with blood pressure, among which, trans-β-carotene had the most substantial contribution (57). Moreover, trans-β-carotene, as a provitamin A, could decrease children’s body mass index (BMI), truncal fat mass, and total body fat mass, while vitamin A exhibited the opposite effects (58). Studies have indicated that increasing serum retinol level is positively associated with a higher prevalence of obesity and other metabolic indicators, including lower HDL-C and higher fasting blood glucose levels (59, 60). Moreover, in this study, the Qgcomp and RCS models showed that vitamin A was positively associated with the risk of MAFLD. This might be because vitamin A could exacerbate high-fat diet-induced hepatic steatosis, which dominates MAFLD patients (61). In addition, vitamin A is mainly dependent on its metabolite retinoic acid, which acts as a transcription factor, activating retinoic acid and retinoid X receptors to improve metabolic disorders (62). Thus, it was hypothesized that various dietary patterns might affect vitamin A, and vitamin A metabolism may be disturbed in patients with MAFLD.

Currently, numerous studies have mainly focused on the specific molecular mechanism of a single vitamin and mineral on NAFLD. For instance, VD3 could increase the levels of the mitochondrial contact site and cristae organizing system (MICOS) 60 by regulating vitamin D receptor (VDR) to ameliorate age-associated NAFLD (63). Vitamin E could activate the AMPK signaling pathway to reduce fatty acid synthesis and decrease oxidative stress (64). Ascorbic acid could activate the FGF21/FGFR2/adiponectin pathway to alleviate hepatocyte stress as well as peroxisome proliferator-activated receptor α (PPARα) and improve the visceral obesity and NAFLD (65). Moreover, studies have demonstrated that vitamin B_12_ and folate could facilitate the β-oxidation of fatty acids and regulate autophagy and inflammation by modifying multiple hepatic proteins to improve non-alcoholic steatohepatitis (66). In summary, a single vitamin and mineral can improve NAFLD by decreasing the level of oxidative stress and inflammation, inhibit the synthesis of fatty acids, and promote the utilization of fatty acids, providing a way forward in the MAFLD research. Moreover, the combined effects of multiple vitamins and minerals on NAFLD or MAFLD are not clear yet. Therefore, further studies are needed to explore it.

The current study has several strengths. First, this study probed into the combined effects of multiple nutrients using various statistical models to circumvent biased results. Second, the weight of individual nutrients on the combined effect was assessed, which helped in determining the relative contents of diverse nutrients in supplementation or dietary intake. Lastly, the data was obtained from NHANES, which strengthens the reliability of the results. However, there were also certain limitations to this study. A cross-sectional study design inherently poses challenges in establishing a causal relationship, necessitating further corroboration through longitudinal cohort studies or clinical trials. Additionally, although adjustments were made for several factors, some potential confounders might have been overlooked. Moreover, a singular measurement might not accurately reflect the long-term nutrient status of participants. Furthermore, this study did not include some crucial vitamins and trace elements, such as vitamin B groups and Zn. Future studies should incorporate a longitudinal follow-up of comprehensive nutrient concentrations and various metabolic indicators. Lastly, while VCTE is extensively used to diagnose liver steatosis and fibrosis due to its high efficiency and non-invasive nature, the cut-off value for diagnosis is still debatable, and liver biopsy retains its position as the gold standard.

## 5 Conclusion

This study suggested that the combination of 15 nutrients, excluding Se, α-tocopherol and γ-tocopherol, was inversely associated with the risk of MAFLD. Simultaneously, this study offered an appropriate compositional ratio. Consequently, it was suggested that the supplementation of multiple vitamins and minerals, either with reduced ratios of Se and two types of vitamin E or entirely without them, might reduce MAFLD prevalence. Nevertheless, additional research is imperative to corroborate these findings.

## Data availability statement

The original contributions presented in the study are included in the article/Supplementary material, further inquiries can be directed to the corresponding author.

## Ethics statement

The studies involving humans were approved by National Center for Health Statistics Ethics Review Board. The studies were conducted in accordance with the local legislation and institutional requirements. The participants provided their written informed consent to participate in this study.

## Author contributions

PG: Formal analysis, Visualization, Writing – original draft. JY: Methodology, Supervision, Writing – review & editing.
