# Supplementary material for: Association of multiple serum minerals and vitamins with metabolic dysfunction-associated fatty liver disease in US adults: National Health and Nutrition Examination Survey 2017–2018
Source: Front Nutr. 2024 Mar 18;11:1335831. doi: 10.3389/fnut.2024.1335831 (PMC10982334; doi:10.3389/fnut.2024.1335831)
Supplement: Supplementary file 1 [file Data_Sheet_1.docx]

**Supplementary Figures**


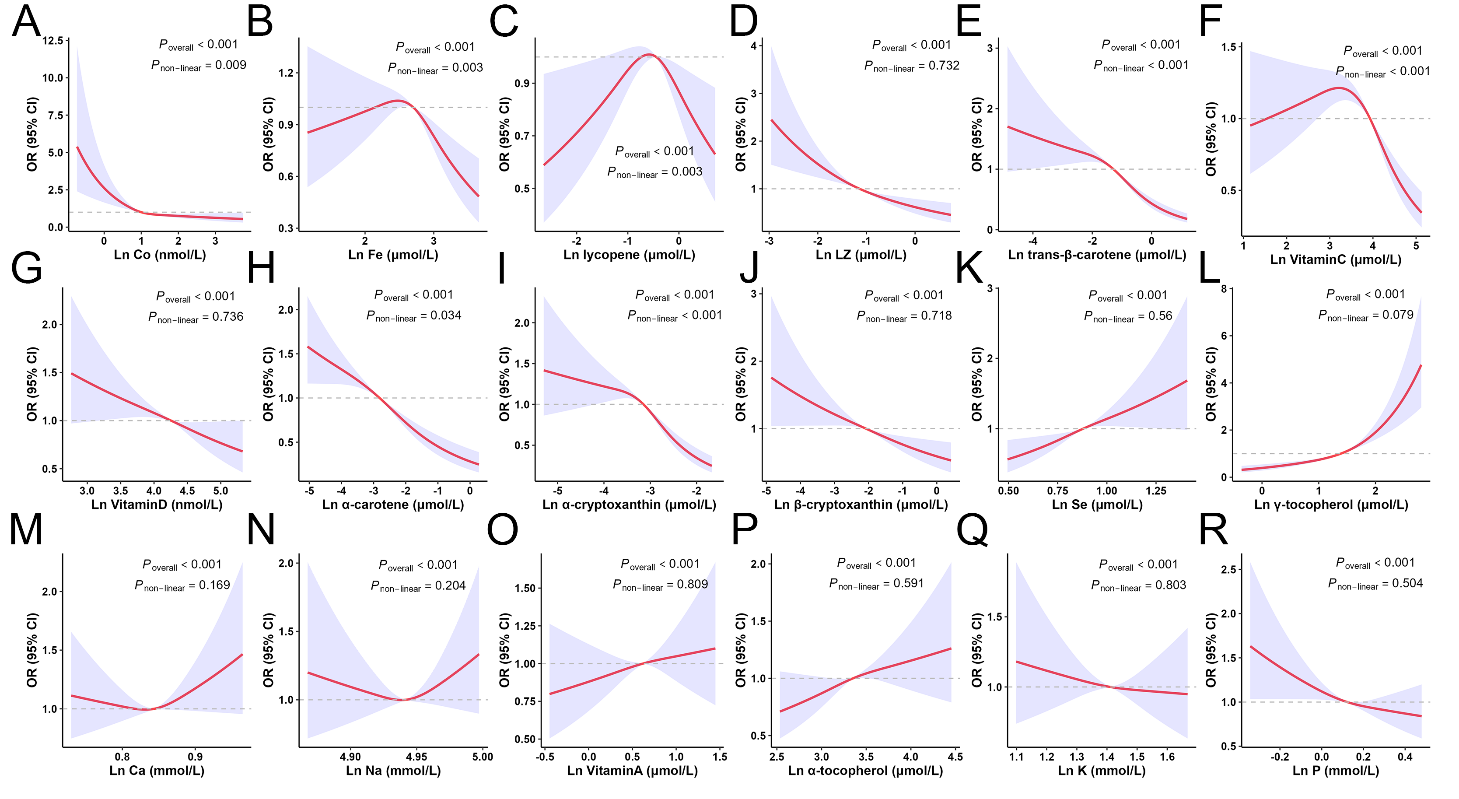


**Figure S1.** The dose-response relationship between the serum concentration of 18 types of nutrients and the risk of MAFLD was evaluated using the restricted cubic spline regression model. The model was adjusted for age, gender, race, education level, smoking status, drinking status, physical activity and poverty income ratio. Notably, the serum concentration was naturally log-transformed.


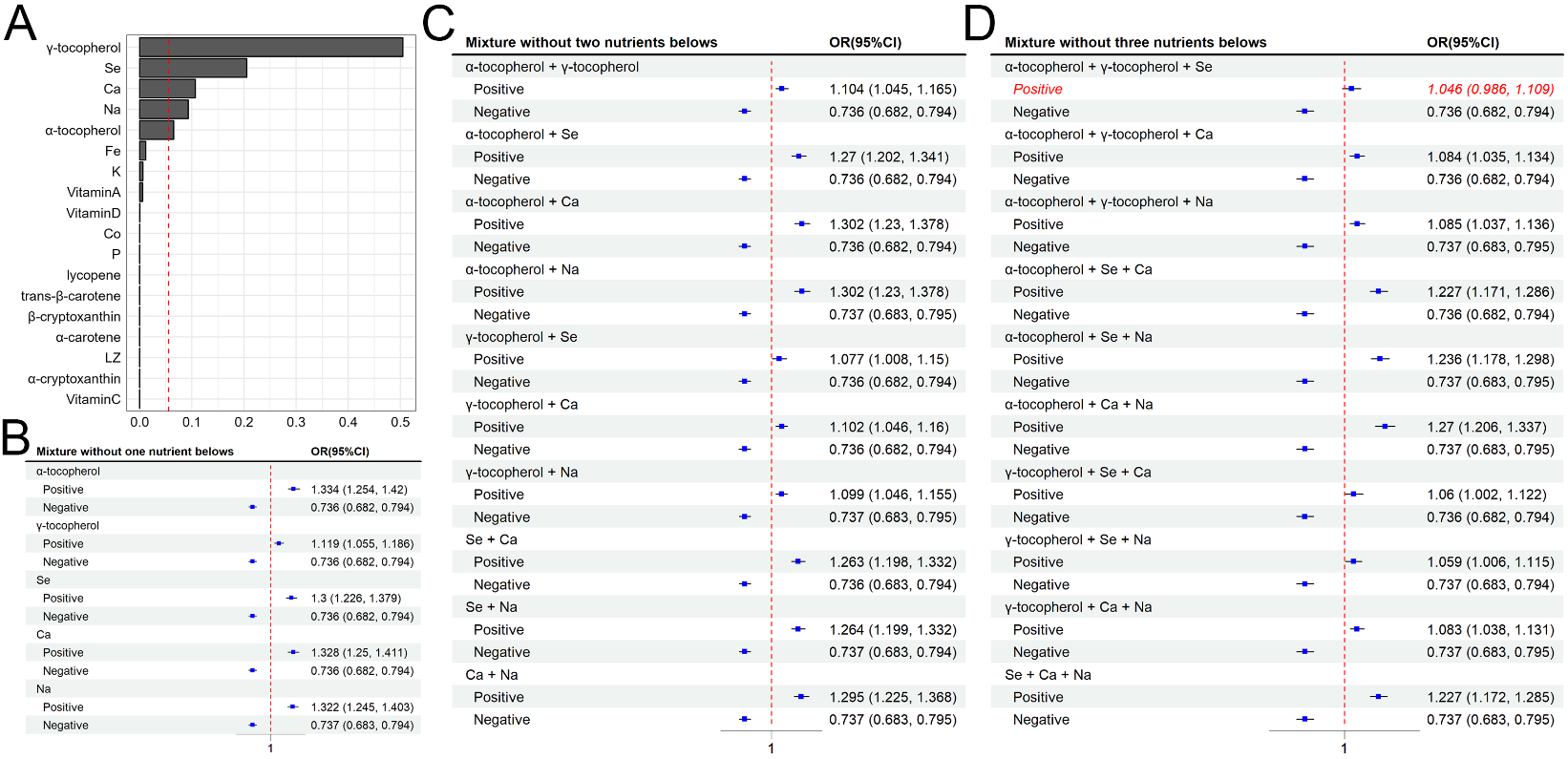


**Figure S2**. The results of WQS regression. (A) The weights of 18 types of nutrients were calculated by WQS regression with positive constrain. The joint effect was assessed by WQS regression after removing any one (B), two (C), or three (D) of α-tocopherol, γ-tocopherol, Se, Ca and Na in turn.

**Supplementary Tables**

Table S1. Logistic regression to assess the effect of a single nutrient on MAFLD.

| Nutrients | Model 1  OR (95% CI) | Model 2  OR (95% CI) |
| --- | --- | --- |
| **P** |  |  |
| Continuous | 0.367 (0.217, 0.616) | 0.461 (0.269, 0.787) |
| Reference | 1 | 1 |
| Q2 | 0.912 (0.742, 1.121) | 0.937 (0.760, 1.156) |
| Q3 | 0.769 (0.611, 0.970) | 0.821 (0.649, 1.041) |
| Q4 | 0.789 (0.650, 0.958) | 0.866 (0.709, 1.059) |
| **K** |  |  |
| Continuous | 1.273 (0.570, 2.840) | 0.687 (0.293, 1.608) |
| Reference | 1 | 1 |
| Q2 | 1.126 (0.907, 1.398) | 1.071 (0.859, 1.335) |
| Q3 | 1.141 (0.921, 1.413) | 1.040 (0.835, 1.295) |
| Q4 | 1.167 (0.956, 1.424) | 1.025 (0.831, 1.264) |
| **Na** |  |  |
| Continuous | 2.809 (0.071, 110.646) | 3.613 (0.085, 152.74) |
| Reference | 1 | 1 |
| Q2 | 0.857 (0.661, 1.113) | 0.845 (0.649, 1.101) |
| Q3 | 0.887 (0.724, 1.087) | 0.901 (0.732, 1.107) |
| Q4 | 1.013 (0.827, 1.240) | 1.015 (0.826, 1.247) |
| **Ca** |  |  |
| Continuous | 1.332 (0.207, 8.575) | 2.968 (0.437, 20.22) |
| Reference | 1 | 1 |
| Q2 | 0.922 (0.746, 1.139) | 0.958 (0.773, 1.188) |
| Q3 | 0.904 (0.730, 1.118) | 0.965 (0.776, 1.199) |
| Q4 | 1.115 (0.910, 1.368) | 1.196 (0.971, 1.474) |
| **Co** |  |  |
| Continuous | 0.661 (0.564, 0.772) | 0.668 (0.568, 0.783) |
| Reference | 1 | 1 |
| Q2 | 0.836 (0.655, 1.063) | 0.823 (0.641, 1.051) |
| Q3 | 0.649 (0.503, 0.835) | 0.643 (0.494, 0.833) |
| Q4 | 0.496 (0.387, 0.634) | 0.503 (0.389, 0.648) |
| **Fe** |  |  |
| Continuous | 0.882 (0.733, 1.059) | 0.779 (0.642, 0.945) |
| Reference | 1 | 1 |
| Q2 | 0.931 (0.753, 1.152) | 0.885 (0.713, 1.099) |
| Q3 | 0.923 (0.747, 1.141) | 0.851 (0.686, 1.057) |
| Q4 | 0.825 (0.668, 1.018) | 0.704 (0.564, 0.877) |
| **Se** |  |  |
| Continuous | 3.474 (1.997, 6.079) | 3.451 (1.957, 6.124) |
| Reference | 1 | 1 |
| Q2 | 1.065 (0.867, 1.310) | 1.055 (0.856, 1.301) |
| Q3 | 1.279 (1.038, 1.576) | 1.271 (1.028, 1.573) |
| Q4 | 1.480 (1.199, 1.827) | 1.456 (1.174, 1.807) |
| **α-tocopherol** |  |  |
| Continuous | 1.352 (1.062, 1.724) | 1.356 (1.051, 1.753) |
| Reference | 1 | 1 |
| Q2 | 1.124 (0.913, 1.384) | 1.111 (0.899, 1.374) |
| Q3 | 1.099 (0.892, 1.356) | 1.112 (0.895, 1.382) |
| Q4 | 1.258 (1.019, 1.553) | 1.249 (1.001, 1.558) |
| **VitaminA** |  |  |
| Continuous | 1.232 (0.961, 1.580) | 1.184 (0.912, 1.537) |
| Reference | 1 | 1 |
| Q2 | 1.095 (0.890, 1.347) | 1.073 (0.868, 1.326) |
| Q3 | 1.186 (0.963, 1.461) | 1.138 (0.917, 1.413) |
| Q4 | 1.190 (0.966, 1.466) | 1.155 (0.928, 1.438) |
| **γ-tocopherol** |  |  |
| Continuous | 2.028 (1.760, 2.342) | 2.311 (1.988, 2.692) |
| Reference | 1 | 1 |
| Q2 | 1.559 (1.269, 1.916) | 1.658 (1.343, 2.049) |
| Q3 | 1.438 (1.175, 1.762) | 1.569 (1.271, 1.939) |
| Q4 | 2.794 (2.247, 3.482) | 3.370 (2.681, 4.249) |
| **α-carotene** |  |  |
| Continuous | 0.782 (0.726, 0.842) | 0.722 (0.665, 0.784) |
| Reference | 1 | 1 |
| Q2 | 0.864 (0.695, 1.074) | 0.755 (0.603, 0.945) |
| Q3 | 0.715 (0.577, 0.886) | 0.600 (0.477, 0.752) |
| Q4 | 0.512 (0.414, 0.632) | 0.396 (0.313, 0.501) |
| **α-cryptoxanthin** |  |  |
| Continuous | 0.676 (0.591, 0.772) | 0.588 (0.505, 0.682) |
| Reference | 1 | 1 |
| Q2 | 0.882 (0.707, 1.099) | 0.842 (0.673, 1.054) |
| Q3 | 0.729 (0.588, 0.903) | 0.661 (0.529, 0.826) |
| Q4 | 0.519 (0.420, 0.642) | 0.413 (0.326, 0.521) |
| **trans-β-carotene** | NA | NA |
| Continuous | 0.688 (0.632, 0.748) | 0.651 (0.593, 0.714) |
| Reference | 1 | 1 |
| Q2 | 0.800 (0.641, 0.997) | 0.746 (0.595, 0.934) |
| Q3 | 0.669 (0.538, 0.829) | 0.601 (0.479, 0.753) |
| Q4 | 0.405 (0.327, 0.501) | 0.350 (0.277, 0.442) |
| **β-cryptoxanthin** | NA | NA |
| Continuous | 0.868 (0.795, 0.947) | 0.793 (0.716, 0.877) |
| Reference | 1 | 1 |
| Q2 | 0.792 (0.640, 0.980) | 0.759 (0.610, 0.943) |
| Q3 | 0.759 (0.613, 0.939) | 0.692 (0.553, 0.865) |
| Q4 | 0.666 (0.539, 0.821) | 0.533 (0.420, 0.675) |
| **LZ** | NA | NA |
| Continuous | 0.683 (0.600, 0.777) | 0.635 (0.552, 0.731) |
| Reference | 1 | 1 |
| Q2 | 0.717 (0.579, 0.889) | 0.660 (0.530, 0.822) |
| Q3 | 0.760 (0.612, 0.943) | 0.690 (0.551, 0.863) |
| Q4 | 0.554 (0.448, 0.684) | 0.494 (0.393, 0.620) |
| **lycopene** |  |  |
| Continuous | 0.983 (0.862, 1.12) | 0.995 (0.866, 1.141) |
| Reference | 1 | 1 |
| Q2 | 1.129 (0.914, 1.394) | 1.110 (0.896, 1.375) |
| Q3 | 0.976 (0.793, 1.202) | 0.956 (0.771, 1.186) |
| Q4 | 0.936 (0.760, 1.151) | 0.946 (0.761, 1.176) |
| **Vitamin C** | NA | NA |
| Continuous | 0.798 (0.716, 0.887) | 0.776 (0.692, 0.869) |
| Reference | 1 | 1 |
| Q2 | 1.008 (0.812, 1.252) | 0.964 (0.772, 1.205) |
| Q3 | 0.830 (0.671, 1.027) | 0.781 (0.625, 0.975) |
| Q4 | 0.539 (0.438, 0.664) | 0.508 (0.407, 0.634) |
| **Vitamin D** | NA | NA |
| Continuous | 0.766 (0.65, 0.902) | 0.736 (0.615, 0.879) |
| Reference | 1 | 1 |
| Q2 | 1.040 (0.841, 1.286) | 0.963 (0.774, 1.199) |
| Q3 | 0.919 (0.745, 1.134) | 0.853 (0.684, 1.065) |
| Q4 | 0.740 (0.601, 0.911) | 0.720 (0.574, 0.902) |

Abbreviations: LZ, lutein+zeaxanthin; OR, odds ratio; 95% CI, 95% confidence interval.

Model 1 was an unadjusted model.

Model 2 was adjusted for age, gender, race, education level, smoking status, drinking status, physical activity and poverty income ratio.

Table S2. The combined effect of 18 types of nutrients.

| Methods | OR (95% CI) |
| --- | --- |
| WQS positive | 1.356 (1.271, 1.447) |
| WQS negative | 0.736 (0.682, 0.794) |
| Qgcomp | 0.964 (0.882, 1.054) |

Abbreviations: WQS: weighted quantile sum; Qgcomp: quantile-g-computation; OR, odds ratio; 95% CI, 95% confidence interval. Both WQS regression and Qgcomp were adjusted for age, gender, race, education level, smoking status, drinking status, physical activity and poverty income ratio.

Table S3. The combined effect of 18 types of nutrients based on complete datasets.

| Methods | OR (95% CI) |
| --- | --- |
| WQS positive | 1.475 (1.348, 1.613) |
| WQS negative | 0.721 (0.663, 0.784) |
| Qgcomp | 0.964 (0.855, 1.086) |

Abbreviations: WQS: weighted quantile sum; Qgcomp: quantile-g-computation; OR, odds ratio; 95% CI, 95% confidence interval. Both WQS regression and Qgcomp were adjusted for age, gender, race, education level, smoking status, drinking status, physical activity and poverty income ratio.

Table S4. The combined effect of 15 types of nutrients based on complete datasets.

| Methods | OR (95% CI) |
| --- | --- |
| WQS positive | 1.035 (0.946, 1.132) |
| WQS negative | 0.721 (0.663, 0.784) |
| Qgcomp | 0.780 (0.706, 0.861) |

Abbreviations: WQS: weighted quantile sum; Qgcomp: quantile-g-computation; OR, odds ratio; 95% CI, 95% confidence interval. Both WQS regression and Qgcomp were adjusted for age, gender, race, education level, smoking status, drinking status, physical activity and poverty income ratio.
